# Maternal diet quality trajectories from pregnancy to 3.5 years postpartum and associated maternal factors

**DOI:** 10.1007/s00394-024-03402-1

**Published:** 2024-05-28

**Authors:** Meaghan J. Sexton-Dhamu, Ewa A. Szymlek-Gay, Katherine M. Livingstone, Li Ming Wen, Miaobing Zheng

**Affiliations:** 1 Institute for Physical Activity and Nutrition, School of Exercise and Nutrition Sciences, Deakin University, 221 Burwood Highway, Geelong, VIC 3125 Australia; 2 School of Public Health and Sydney Medical School, The University of Sydney, Sydney, Australia; 3 Health Promotion Unit, Population Health Research and Evaluation Hub, Sydney Local Health District, Sydney, NSW Australia

**Keywords:** Diet quality, Group-based trajectory modelling, Maternal, Pregnancy, Postpartum, Maternal factors

## Abstract

**Purpose:**

This study examined maternal diet quality trajectories from pregnancy to 3.5 years postpartum and associated maternal factors.

**Methods:**

Data of 473 Australian women from the Healthy Beginnings Trial were used. A food frequency questionnaire collected dietary intake in pregnancy and 1, 2 and 3.5 years postpartum. Diet quality scores were calculated using the 2013 Dietary Guideline Index (DGI-2013) and RESIDential Environments Guideline Index (RDGI). Group-based trajectory modelling identified diet quality trajectories from pregnancy to 3.5 years postpartum. Multivariable logistic regression investigated factors associated with maternal diet quality trajectories.

**Results:**

Two stable trajectories of low or high diet quality were identified for the DGI-2013 and RDGI. Women who smoked had higher odds of following the low versus the high DGI-2013 (OR 1.77; 95%CI 1.15, 2.75) and RDGI (OR 1.80; 95%CI 1.17, 2.78) trajectories, respectively. Women who attended university had lower odds of following the low versus the high DGI-2013 (OR 0.41; 95%CI 0.22, 0.76) and RDGI (OR 0.38; 95%CI 0.21, 0.70) trajectories, respectively. Women who were married had lower odds of following the low versus the high DGI-2013 trajectory (OR 0.39; 95%CI 0.17, 0.89), and women who were unemployed had higher odds of following the low versus the high RDGI trajectory (OR 1.78; 95%CI 1.13, 2.78). Maternal age, country of birth, household composition and pre-pregnancy body mass index were not associated with diet quality trajectories.

**Conclusion:**

Maternal diet quality trajectories remained stable from pregnancy to 3.5 years postpartum. Women who smoked, completed high school or less, were not married or were unemployed tended to follow low, stable diet quality trajectories.

**Supplementary Information:**

The online version contains supplementary material available at 10.1007/s00394-024-03402-1.

## Introduction

Healthy eating behaviours are vital during pregnancy and postpartum, as a good maternal diet has been linked with positive health outcomes for both mothers and their children [1]. The Australian Dietary Guidelines recommend eating a healthful diet consisting of a diverse variety of foods from five food groups (e.g., vegetables (including legumes and beans), fruit, grain (cereal foods), lean meats and alternatives and dairy products and alternatives) [2]. In Australia, most adults do not meet these recommendations; for example, 49% of Australian adults do not eat the recommended two serves of fruit, and 92% do not eat the recommended five to six serves of vegetables [3]. Moreover, prior research has shown that 34% and 90% of Australian pregnant women did not meet the ADG recommendations for fruits and vegetables, respectively, and almost 84% ate up to 2.5 serves of discretionary foods [4], which are energy-dense and nutrient-poor foods that are high in saturated fats, sugars, salt and/or alcohol [2]. Insufficient nutrition in utero has been shown to substantially alter the developmental trajectory of the foetus and result in reprogramming of the epigenome, leading to adverse outcomes such as obesity and cardiovascular diseases in adulthood [5]. It is, therefore, essential that women maintain a high-quality diet during this period to promote good maternal health and the lifelong health of their child.

A priori diet quality indices are widely used to assess adherence to national dietary recommendations [6]. The 2013 Dietary Guideline Index (DGI-2013) is a food-based index comprising encouraged and discouraged components based on age- and sex-specific recommendations of the Australian Dietary Guidelines [7]. The DGI-2013 has been applied to both brief [8] and long [9] dietary assessment tools. Similarly, the RESIDential Environments Dietary Guideline Index (RDGI) is food-based; however, the scoring is based on simple dietary questionnaire items, allowing it to be more readily applied to brief dietary assessment tools [10]. Both indices have been widely used in Australian adults aged 18–50 years [7–10], and both have been used to cross-sectionally investigate diet quality in women [8, 10]. Thus, the two indices were selected for use in pregnant women with pregnancy-specific cut-offs. To date, only one study has explored longitudinal changes in maternal diet quality (i.e., trajectories) using posterior or ‘data-driven’ principal component analysis; the authors reported maternal diet quality trajectories were stable from pre-pregnancy to 8–9 years postpartum [11]. Whether these associations differ by the diet quality index used has not been assessed.

Several factors have been shown to be associated with diet quality. For example, a recent systematic review highlighted that women with low educational attainment and income levels were more likely to have a poor diet quality consisting of high-fat foods and sugar-sweetened beverages [12] than women with high educational attainment and income levels. A study of 1325 pregnant women from the United States identified that those who were younger, single or who smoked had lower Healthy Eating Index (HEI) scores [13]. Another study of 527 Australian women reported that women with a university degree or higher had a significantly better DGI score at 4 months postpartum than women with a lower education level [14]. Thus, understanding which women have a poor diet quality in pregnancy and postpartum is important so that interventions to improve their eating behaviours can be tailored to their needs.

To support efforts to improve healthy eating in women and the long-term health outcomes of women and their children, it is critical to identify and characterise population groups most at risk of unhealthy eating behaviours. However, limited studies have investigated maternal factors associated with longitudinal diet quality trajectories [11] and none in Australian women. Examining factors associated with poor maternal diet quality using trajectory modelling approaches may help identify at-risk populations. Therefore, this study aimed to assess overall maternal diet quality trajectories from pregnancy to 3.5 years postpartum using two diet quality indices and identify associated maternal factors.

## Methods

### Study design and participants

This was a secondary analysis of longitudinal data from the Healthy Beginnings Trial, a home-based early childhood obesity prevention intervention involving first-time mothers and their children. Detailed information on the randomised controlled trial and results has been previously published [15–17]. Briefly, the trial consisted of two phases, a two-year intervention phase conducted from 2007 to 2010 and a three-year follow-up phase from 2011 to 2014. The intervention group received eight home visits from a trained early childhood nurse, consisting of one antenatal visit at 30–36 weeks gestation and seven postnatal visits starting from birth to 24 months [17, 18]. Nurses promoted healthy infant feeding, child and family nutrition and physical activity and social support; these visits were complemented with proactive telephone support. The control group received usual care.

Pregnant women who attended one of two metropolitan antenatal clinics in Western Sydney were invited to participate in the trial. Women were eligible to participate if they were at least 16 years old, were 24–34 weeks pregnant, they or their guardian could communicate in English, were local residents and could provide informed consent. Women were excluded if, after giving birth, their infant was diagnosed with a medical condition affecting physical activity, eating behaviours, weight or height/length. Ethics approval was obtained from the Sydney South West Area Health Service ethics review committee (RPAH Zone, Protocol No X10-0312 and HREC/10/RPAH/546) and the Human Ethics Advisory Group—Health at Deakin University (HEAG-H 194_2021). Informed consent was obtained from all participants. This study is reported according to the Strengthening the Reporting of Observational Studies in Epidemiology (STROBE) guidelines (Supplementary Table 1) [19].

### Dietary intake

Women’s dietary data were collected at baseline (24–34 weeks gestation) and at 1, 2 and 3.5 years postpartum. Maternal dietary intake was collected using a 15-item semi-quantitative food frequency questionnaire (FFQ) through face-to-face interviews with a trained nurse at all time points [19]. This questionnaire was based on dietary questions from the New South Wales Population Health Survey Australia [16, 21] and has been previously validated [22]. The FFQ collected information on frequency of intake of vegetables, fruit, bread, breakfast cereal, cooked grains/cereals, milk, processed meat, takeaway meals/snacks, potato products, sugar-sweetened beverages, fruit juice and water [16, 21]. Women were asked to answer how many times/serves per day, week or month they consumed the food and beverage items or whether they were rarely or not consumed (Supplementary Table 2).

### Diet quality

Diet quality was calculated using modified versions of the DGI-2013 and the RDGI, which assess adherence to the 2013 Australian Dietary Guidelines [7, 10]. The DGI-2013 was chosen because it is a commonly used index with food-based components designed to score dietary intakes according to national recommendations [7] and can be applied to brief dietary assessment tools [8]. The RDGI was selected because the dietary questions from the questionnaire could be directly applied to the indicators of the index.

The DGI-2013 is a food-based score developed to assess adherence to age- and sex-specific recommendations of the 2013 Australian Dietary Guidelines [7] and has been shown to be a valid measure of diet quality in the Australian population [7, 9, 23, 24]. The original DGI-2013 comprises 13 components consisting of seven encouraged (i.e., diet variety, vegetables, fruit, grains/cereals, lean meat and alternatives, dairy and alternatives and fluid intake) and six discouraged (i.e., discretionary foods, saturated fat, unsaturated fat, added salt, added sugar and alcohol) components to give a total score ranging from 0 to 130 [7]. The DGI-2013 was adapted for use in the present study based on the dietary data available. The modified DGI-2013 consisted of eight components, six of which were scored out of 10 (i.e., vegetables, fruit, dairy and alternatives, fluid intake, discretionary foods and added sugar), and two of which were scored out of 5 (i.e., grains/cereals and saturated fat; Supplementary Table 3). Discouraged components were reverse scored. Scoring was proportional, with a maximum score indicating that the national recommendations had been met. Questions on diet variety, type of bread consumed, lean meat and alternatives intake, whether fat was trimmed from meat, salt intake and alcohol intake were excluded. The total score for the modified DGI-2013 ranged from 0 to 70, with a higher score indicating better diet quality.

The RDGI is a food-based score that assesses adherence to the 2013 Australian Dietary Guidelines recommendations in adults >18 years [10]. The original version of the RDGI contains 10 components comprising six encouraged (i.e., vegetables, fruit, grains/cereals, lean meats, dairy and alternatives and fluid intake) and four discouraged (i.e., saturated fat, added salt, added sugar and alcohol) components. The original RDGI score ranges between 0 and 100 [10]. Similarly, the RDGI was modified to suit the dietary intake data in the present study, resulting in seven components (Supplementary Table 4). This included three components (i.e., vegetables, fruit, fluid intake) scored out of 10, one component (i.e., grains/cereals) scored out of 7.5, one component (i.e., saturated fat) scored out of 6 and two components (i.e., dairy and alternatives and added sugar) scored out of 5. Discouraged components were reverse scored. Scoring was proportional, with a maximum score for a component suggesting that national recommendations had been met. The components in the original RDGI on intakes of lean meats, cheese, hot drinks (e.g., tea and coffee), salt, foods high in added sugars (e.g., biscuits, cakes and chocolate) and alcohol, as well as how often fat was trimmed from meat and the type of bread consumed, were excluded. The total score for the modified RDGI ranged between 0 and 53.5, with a higher score suggesting a better diet quality.

### Background characteristics

Background data of women were collected at baseline. Women reported their age (years), country of birth (Australia versus other), annual gross household income (<AUD$40,000, $40,000–80,000 and >$80,000) [16, 20], current employment status (employed versus unemployed) [16, 20], highest completed qualification (high school or less versus trade certificate/diploma versus university degree or higher) [16, 20], marital status (married versus not married), household composition (two-parent household versus other), smoking status (non-smoker versus past/current smoker) and pre-pregnancy weight and height without shoes. Past and current smokers were grouped, as prior research indicates that former smokers have similar dietary behaviours to current smokers [25]. The World Health Organization’s classification of overweight or obesity was used to categorise women’s pre-pregnancy body mass index (BMI; kg/m^2^) as underweight/normal weight (BMI < 25.0 kg/m^2^) and overweight/obese (BMI ≥ 25.0 kg/m^2^) [26].

### Statistical analysis

Group-based trajectory modelling (GBTM) was used to estimate diet quality trajectory groups from 24 to 34 weeks gestation to 3.5 years postpartum using the traj command in Stata version 17.0 [27]. To facilitate comparison between the two diet quality indices, the raw DGI-2013 and RDGI scores were standardised (z-scores) before identifying the diet quality trajectories. GBTM identifies heterogeneous groups of women following similar diet quality trajectories within a study population and estimates the proportion of women in each group and their probability of membership to that group over time [28, 29]. Women with two or more diet quality data over four time points (median = 3) were included in the analyses to identify the diet quality trajectories. Mean gestational age in months was used as the time point variables, with baseline (i.e., 24–34 weeks gestation) coded as 0 and 1, 2 and 3.5 years postpartum coded as 12, 24 and 42 months, respectively. Censored normal models specifying the cubic function of time (in months) as the independent variable and repeated measurements of diet quality z-scores as the outcome variable were performed. Models with 2–5 groups were conducted and compared using the following criteria: Bayesian information criterion (BIC), entropy (> 0.7), the minimum proportion of women (> 5% of total sample) assigned to each trajectory group and model parsimony [29, 30]. The final model was chosen based on higher entropy and a higher BIC (less negative), which indicate a better model fit, clinical interpretability and model parsimony, in which a simpler model with greater interpretability is preferred [29, 30]. Once the optimal number of groups was chosen, the optimal shape of each trajectory was tested using various polynomial functions (i.e., linear, quadratic and cubic) of the time points of diet quality assessment [29, 30]. Significant polynomial functions were retained [29, 30].

Descriptive analyses were conducted to summarise cohort characteristics by the identified diet quality trajectories. Analyses were conducted to assess differences in diet quality scores by intervention allocation (intervention versus control group). There were no differences by intervention allocation at all time points except for RDGI score at 2 years (mean difference −1.20; 95%CI −2.35, −0.06) (Supplementary Table 5). A 1.2-point difference in RDGI score was considered small given that the overall RDGI score is 53.5. Therefore, the intervention and control groups were pooled for the present analysis. Intervention allocation was included as a covariate in all analyses to account for potential differences between groups.

Multivariable logistic regression was used to assess the association between maternal factors (maternal age, country of birth, educational attainment, marital status, household composition and smoking status) and the identified diet quality trajectory groups. First, univariable logistic regression was conducted with each maternal factor as the exposure and the low diet quality trajectory groups as the outcome; the high diet quality trajectory group was the reference group in all analyses. All maternal factors were then included in the multivariable model. Pearson’s correlation was undertaken to test for multicollinearity among the maternal factors (Supplementary Table 6). Household income was removed from the analysis due to data missingness (*n* = 41, 8.5%) and high correlation with employment status (*r* = –0.51). Maternal pre-pregnancy BMI was included in sensitivity analyses due to missing data (*n* = 26, 5.4%) (Supplementary Fig. 1).

In addition, *t*-tests or χ^2^ tests for continuous and categorical variables, respectively, were also performed to compare cohort characteristics between included and excluded participants.

### Sensitivity analyses

To examine the effect of missing maternal factors on the analysis, multiple imputations by chained equation with 10 datasets were conducted to impute the missing maternal factors (Supplementary Fig. 1). Linear and logistic regressions were used to predict the missing continuous and categorical maternal factors, respectively, and the ‘mi estimate’ Stata command was then used to pool estimates from the 10 datasets.

Additional sensitivity analyses were conducted using raw DGI-2013 and RDGI diet quality scores as the outcome to identify the diet quality trajectory groups and associated maternal factors. Further analyses were conducted to investigate associations between pre-pregnancy BMI, along with other maternal factors, and DGI-2013 and RDGI trajectories. All analyses were performed using Stata 17.0. Results were considered significant at *p* < 0.05. Results are reported as odds ratio (OR) and 95% confidence interval (95%CI).

## Results

Of the 667 women who participated in the Healthy Beginnings Trial at baseline, 23 were excluded for being <18 years old. This exclusion allowed for comparison between the DGI-2103 and RDGI because the RDGI was developed for use in adults aged >18 years [10]. A further 159 women were excluded for having only one time point of dietary assessment. Therefore, 485 women (intervention [*n* = 253] versus control [*n* = 232] group) were included in the trajectory modelling to identify the DGI-2013 and RDGI trajectories. An additional 12 women were excluded from the primary analysis for having missing data for maternal factors (Supplementary Fig. 1). There was a statistically significant difference between included and excluded participants in cohort characteristics (Supplementary Table 7). The excluded women were more likely to be younger, unmarried and not live in a two-parent household and have lower educational attainment and higher rates of being unemployed. In addition, the excluded women had, on average, lower DGI-2013 and RDGI diet quality scores at all time points.

### Participant characteristics

Table 1 presents the baseline characteristics of the 485 women. The mean age of women was 27.1 (SD 5.3) years. Most women were born in Australia, were employed, were married, lived in a two-parent household, were a non-smoker and had a BMI of <25 kg/m^2^. In addition, almost one-third of women had completed university, and almost half had an annual household income of >AUD80,000.

**Table 1:** Baseline characteristics of mothers from the Healthy Beginnings Trial (*n* = 485) by diet quality trajectory groups

Characteristic	Overall	DGI-2013 trajectory group		RDGI trajectory group	
	n (%)/mean ± SD/median (IQR)	Low n (%)/mean ± SD/median (IQR)	High n (%)/mean ± SD/median (IQR)	Low n (%)/mean ± SD/median (IQR)	High n (%)/mean ± SD/median (IQR)
n	485	164	321	184	301
Age at baseline, y	27.1 ± 5.3	25.8 ± 5.2	27.9 ± 5.1	25.8 ± 5.4	28.0 ± 5.0
Country of birth, n (%)					
Australia	309 (63.7)	122 (74.4)	187 (58.3)	132 (71.3)	177 (58.8)
Other	174 (35.9)	42 (25.6)	132 (41.1)	51 (27.7)	123 (40.9)
Missing	2 (0.4)	0 (0)	2 (0.6)	1 (0.5)	1 (0.3)
Educational attainment, n (%)					
High school or less	166 (34.2)	77 (47.0)	89 (27.7)	86 (46.7)	80 (26.6)
TAFE certificate/diploma	185 (38.3)	66 (40.2)	119 (37.1)	74 (40.2)	111 (36.9)
University	134 (27.6)	21 (12.8)	113 (35.2)	24 (13.0)	110 (36.5)
Annual household income, n (%)					
<AUD40,000	80 (16.5)	44 (26.8)	36 (11.2)	52 (27.7)	28 (9.3)
AUD40,000–80,000	159 (32.8)	50 (30.5)	109 (34.0)	59 (32.1)	100 (33.2)
>AUD80,000	205 (42.3)	52 (31.7)	153 (47.7)	54 (29.3)	151 (50.2)
Missing	41 (8.4)	18 (11.0)	23 (7.2)	19 (10.3)	22 (7.3)
Employment status, n (%)					
Employed	290 (59.8)	86 (52.4)	204 (63.6)	90 (48.9)	200 (66.4)
Unemployed	194 (40.0)	77 (47.0)	117 (36.4)	93 (50.5)	101 (33.6)
Missing	1 (0.2)	1 (0.6)	0 (0)	1 (0.5)	0 (0)
Marital status					
Married/de facto	46 (9.5)	134 (81.7)	305 (95.0)	154 (83.7)	285 (94.7)
Not married	439 (90.5)	30 (18.3)	16 (5.0)	30 (16.3)	16 (5.3)
Household composition					
Two-parent household	356 (73.4)	112 (68.3)	244 (76.0)	124 (67.4)	232 (77.1)
Other	123 (25.4)	47 (28.7)	76 (23.7)	55 (30.0)	68 (22.6)
Missing	6 (1.2)	5 (3.0)	1 (0.3)	5 (2.7)	1 (0.3)
Smoking status					
Non-smoker	324 (66.8)	89 (54.3)	235 (73.2)	101 (54.9)	223 (74.1)
Current/past smoker	158 (32.6)	74 (45.1)	84 (26.2)	81 (44.0)	77 (25.6)
Missing	3 (0.6)	1 (0.6)	2 (0.6)	2 (1.1)	1 (0.3)
Pre-pregnancy BMI, kg/m^2^	24.0 (21.1, 27.7)	24.7 (21.1, 29.0)	23.9 (21.1, 27.4)	24.3 (21.0, 28.7)	23.9 (21.2, 27.4)
Pre-pregnancy BMI category, n (%)					
Underweight/normal weight	273 (56.3)	82 (50.0)	191 (59.5)	94 (51.1)	179 (59.5)
Overweight/obese	186 (38.3)	69 (42.1)	117 (36.4)	75 (40.8)	111 (36.9)
Missing	26 (5.4)	13 (7.9)	13 (4.0)	15 (8.1)	11 (3.6)

BMI, body mass index; IQR, interquartile range; SD, standard deviation

### Diet quality trajectories

**Fig. 1:**
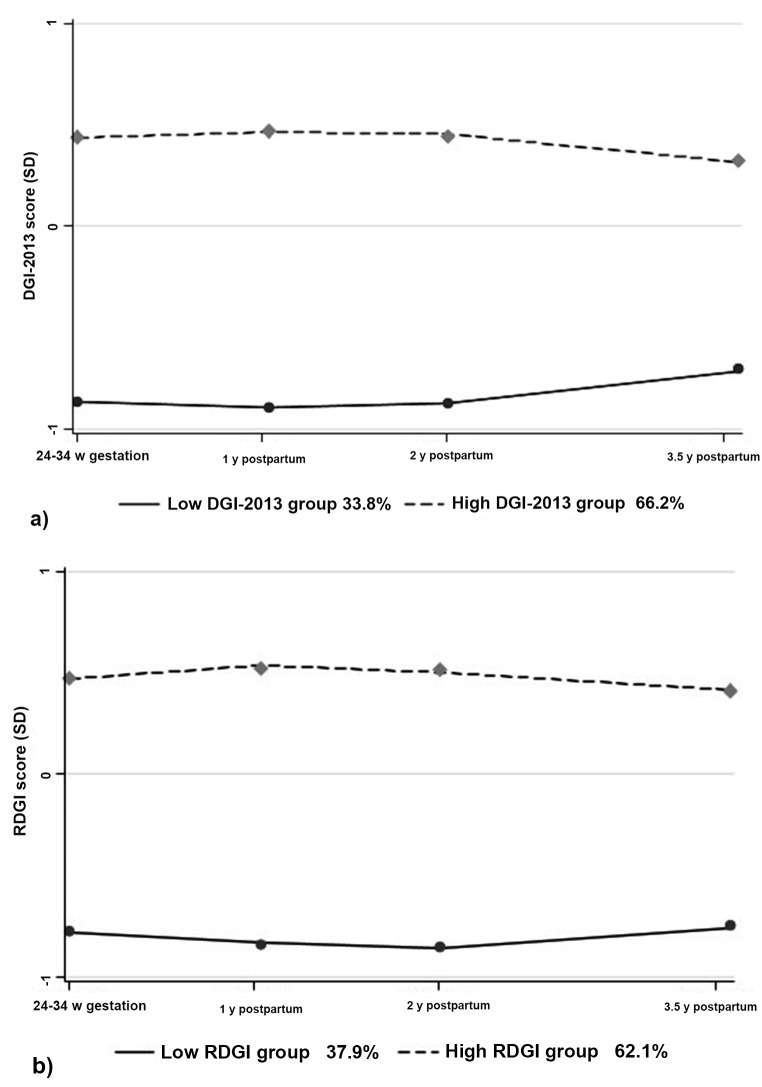
Maternal diet quality z-score trajectories from pregnancy (24–34 weeks gestation) to 3.5 years postpartum for (**a**) 2013 Dietary Guideline Index (DGI-2013) [5] and (**b**) RESIDential Environments Dietary Guideline Index (RDGI) [8]. Notes. DGI-2013, 2013 Dietary Guideline Index; RDGI, RESIDential Environments Dietary Guideline Index; SD, standard deviation; w, weeks; y, year(s)

GBTM identified two diet quality trajectory groups for each of the maternal DGI-2013 and RDGI z-scores from 24 to 34 weeks gestation to 3.5 years postpartum (Fig. 1). Detailed model fit statistics for the DGI-2013 and RDGI z-scores with 2–5 trajectory groups are provided in Supplementary Table 8. Although the three-class model showed a better BIC, the two-class model was ultimately chosen based on clinical interpretability and model parsimony [31]. The DGI-2013 trajectories were characterised as low (*n* = 164, 33.8%) or high (*n* = 321, 66.2%); similarly, the RDGI trajectories were characterised as low (*n* = 184, 37.9%) or high (*n* = 301, 62.1%). Figure 1 shows that the DGI-2013 and RDGI trajectories remained relatively stable from pregnancy to 3.5 years postpartum, with only subtle fluctuations in diet quality (Table 2). This is reflected in the overall mean raw DGI-2013 and RDGI scores from pregnancy to 3.5 years postpartum, which ranged from 43.9 (SD 9.9) to 47.8 (SD 9.2) and 34.9 (SD 7.0) to 36.2 (SD 6.1), respectively (Table 2). DGI-2013 and RDGI scores were lowest at 1 year postpartum and highest in pregnancy. There was up to an almost 10-point and 14-point difference between average scores on the low and high RDGI and DGI-2013 trajectories, respectively, from pregnancy to 3.5 years postpartum, with the largest difference being observed at 1 year postpartum.

**Table 2:** Modified 2013 Dietary Guideline Index (DGI-2013) [5] and RESIDential Environments Dietary Guideline Index (RDGI) [8] scores from 24–34 weeks gestation to 3.5 years postpartum for women in the Healthy Beginnings Trial (*n* = 485)

Diet component	24–34 weeks gestation Mean (SD)	1 year postpartum Mean (SD)	2 years postpartum Mean (SD)	3.5 years postpartum Mean (SD)
DGI-2013				
Overall DGI-2013 score	47.8 (9.2)	43.9 (9.9)	45.6 (8.7)	46.1 (7.2)
Low DGI-2013 trajectory	39.4 (8.0)	34.8 (8.0)	37.7 (7.6)	40.9 (7.3)
High DGI-2013 trajectory	52.1 (6.4)	48.7 (7.1)	50.0 (6.1)	48.4 (5.7)
RDGI				
Overall RDGI score	36.2 (6.1)	34.9 (7.0)	36.0 (6.4)	35.7 (5.7)
Low RDGI trajectory	31.3 (4.9)	28.8 (5.4)	30.4 (5.4)	31.4 (5.1)
High RDGI trajectory	39.2 (4.7)	38.7 (4.7)	39.3 (4.1)	38.1 (4.5)

DGI-2013, 2013 Dietary Guideline Index; RDGI, RESIDential Environments Dietary Guideline Index; SD, standard deviation

#### Maternal factors associated with diet quality trajectory groups

Table 1 describes the characteristics of women following the low or high DGI-2013 and RDGI trajectories. Compared with women from the high DGI-2013 trajectory, more women from the low DGI-2013 trajectory were born in Australia, had completed high school or less, had an annual household income of <AUD40,000, were unemployed, were not married or were a current or past smoker. Similar results were found for the RDGI.

Table 3 shows the results of logistic regression models of the associations between maternal factors and following the low versus the high DGI-2013 or RDGI-2013 trajectory groups. In the univariable model, compared with women more likely to follow the high DGI-2013 trajectory, women were more likely to follow the low DGI-2013 trajectory if they were born in Australia (OR 2.01; 95%CI 1.32, 3.06), were unemployed (OR 1.65; 95%CI 1.12, 2.44) or smoked (OR 2.38; 95%CI 1.59, 3.56) than women who were born in a country other than Australia, employed or a non-smoker. In addition, women who were older (OR 0.92; 95%CI 0.89, 0.96), had attended university (OR 0.23; 95%CI 0.13, 0.40) or were married (OR 0.23; 95%CI 0.12, 0.46) had lower odds of following the low versus the high DGI-2013 trajectory compared with women who were younger, had completed high school or less or who were not married. When all variables were input into the multivariable regression model, educational attainment, marital status and smoking status remained significant and associations were in the same direction; however, the OR effect size for smoking status (OR 1.77; 95%CI 1.15, 2.75) was attenuated, while for educational attainment (OR 0.41; 95%CI 0.22, 0.76) and marital status (OR 0.39; 95%CI 0.17, 0.89), they were strengthened. Comparable results were found in the univariable and multivariable models for the RDGI; however, women who were unemployed had 78% higher odds (OR 1.78; 95%CI 1.13, 2.78) of following the low versus the high RDGI trajectory, and no significant association was found for marital status.

**Table 3:** Univariable and multivariable logistic regression analyses estimating associations between maternal factors and following the low versus the high 2013 Dietary Guideline Index (DGI-2013) [5] or RESIDential Environments Dietary Guideline Index (RDGI) [8] trajectory among pregnant women in the Healthy Beginnings Trial (*n* = 485)

Characteristic	Univariable model			Multivariable model			Univariable model			Multivariable model		
	High DGI-2013 trajectory	Low DGI-2013 trajectory		High DGI-2013 trajectory	Low DGI-2013 trajectory		High RDGI trajectory	Low RDGI trajectory		High RDGI trajectory	Low RDGI trajectory	
	Ref.	OR ^a^ (95% CI)	p	Ref.	OR ^a^ (95% CI)	p	Ref.	OR ^a^ (95% CI)	p	Ref.	OR ^a^ (95% CI)	p
Age, y		0.92 (0.89, 0.96)	< 0.0001		0.96 (0.92, 1.01)	0.12		0.92 (0.89, 0.96)	< 0.0001		0.97 (0.92, 1.01)	0.13
Country of birth, n (%)	1			1			1			1		
Other (Ref.)												
Australia		2.01 (1.32, 3.06)	0.001		1.40 (0.87, 2.26)	0.17		1.81 (1.21, 2.71)	0.004		1.40 (0.87, 2.23)	0.16
Educational attainment, n (%)	1			1			1			1		
High school or below (Ref)												
TAFE certificate/diploma		0.65 (0.42, 1.01)	0.058		0.83 (0.52, 1.34)	0.45		0.65 (0.42, 1.00)	0.048		0.85 (0.53, 1.36)	0.50
University		0.23 (0.13, 0.40)	< 0.0001		0.41 (0.22, 0.76)	0.005		0.21 (0.12, 0.36)	< 0.0001		0.38 (0.21, 0.70)	0.002
Employment status, n (%)	1			1			1			1		
Employed (Ref.)												
Unemployed		1.65 (1.12, 2.44)	0.012		1.25 (00.79, 1.98)	0.34		2.17 (1.48, 3.19)	< 0.0001		1.78 (1.13, 2.78)	0.012
Marital status	1			1			1			1		
Not married (Ref.)												
Married/de facto		0.23 (0.12, 0.46)	< 0.0001		0.39 (0.17, 0.89)	0.025		0.29 (0.15, 0.57)	< 0.0001		0.61 (0.27,1.40)	0.24
Household composition	1			1			1			1		
Other (Ref.)												
Two-parent household		0.70 (0.46, 1.08)	0.110		1.26 (0.73, 2.17)	0.42		0.67 (0.44, 1.02)	0.063		1.12 (0.66, 1.90)	0.68
Smoking status	1			1			1			1		
Non-smoker (Ref.)												
Current/past smoker		2.38 (1.59, 3.56)	< 0.0001		1.77 (1.15, 2.75)	0.01		2.40 (1.62, 3.57)	< 0.0001		1.80 (1.17, 2.78)	0.007

*Note*. Univariable regression models were run for all demographic correlates. All correlates were included in the multivariable model, adjusted for intervention allocation. ^a^ Univariable logistic regression was used to assess the associations between each demographic variable and membership to the low versus the high diet quality trajectory. CI, confidence interval; DGI-2013, 2013 Dietary Guideline Index; OR, odds ratio; RDGI, RESIDential Environments Dietary Guideline Index

For both the DGI-2013 and RDGI, maternal age, country of birth and household composition were not associated with DGI-2013 or RDGI trajectories in the multivariable model.

### Sensitivity analyses

Sensitivity analyses using the raw DGI-2013 and RDGI scores (Supplementary Fig. 2) showed results comparable with those of the DGI-2013 and RDGI z-scores analysis (Supplementary Table 9). Multiple imputations of the missing maternal factors (*n* = 1 to 26 missing, 0.2–5.4%) slightly weakened the effect sizes for most variables in both the DGI-2013 and RDGI univariable and multivariable models; however, the associations were in the same direction (Supplementary Table 10). Pre-pregnancy BMI and associations with following the low versus the high DGI-2013 and RDGI trajectories were examined, and no associations were found (data not shown).

## Discussion

This is the first study, to our knowledge, to have applied a GBTM approach to describe the changes in diet quality of Australian women from pregnancy (24–34 weeks gestation) to 3.5 years postpartum and identify associated maternal factors. The main findings of this study suggest that women’s diet quality remained relatively stable from pregnancy to 3.5 years postpartum, with subtle changes in diet quality. Women who smoked or completed high school or less were more likely to have enduring poor diet quality regardless of the diet quality index used. In addition, women who were unemployed were more likely to follow a low rather than a high RDGI trajectory, while women who were married or in a de facto relationship were less likely to follow a low than a high DGI-2013 trajectory. These findings highlight the importance of establishing healthy dietary behaviours in pregnancy, particularly in women at high risk of having a poor diet quality.

The findings from the present study showed that diet quality trajectories remained relatively stable from 24 to 34 weeks gestation to 3.5 years postpartum, with subtle fluctuations in diet quality, particularly at 1 year postpartum. A United Kingdom study of 2963 women found similar diet quality trajectories from pre-pregnancy to 8–9 years postpartum but identified five trajectory groups [11] compared with the two in this study. This could be due to differences in the analysis of dietary intake, as that study used principal component analysis, a posterior data-driven approach, to derive the diet quality scores versus the a priori method used in our study. Similar findings were reported in a longitudinal study of 110 women from Germany [32] and a small Canadian study of 23 women [33]. In contrast, an Australian study of 301 Australian women with overweight or obesity reported a decrease in HEI scores from early pregnancy (10–20 weeks gestation) to 4 months postpartum [34]. In the current study, all the women were nulliparous. Therefore, the contrasting findings could be because of the higher proportion of multiparous women in that sample, which has been shown to be associated with poor diet quality in pregnancy [35]. Another Australian study using data from a national health survey undertaken in 2011–2012 reported the DGI-2013 scores of women aged 19–50 [36]. However, we cannot directly compare our DGI-2013 scores with those reported from that study because we modified the original DGI scoring method to suit our FFQ and that study did not report DGI-2013 scores for pregnant and postpartum women. There was a substantial difference in diet quality scores between the low and high DGI-2013 and RDGI trajectories, with an almost 14-point difference observed for the DGI-2013 at 1-year postpartum. Similar findings were observed for the RDGI. A study of 19,582 Danish women [37] and a recent study of 1362 Finnish women [38] reported similar wide gaps in mean HEI scores in pregnant women with the lowest diet quality versus those with the highest diet quality. Regarding meeting national recommendations [2], the 14-point difference in DGI-2013 scores in our study suggests that women following the low DGI-2013 trajectory could be missing out on, for example, two food groups, such as eating no serves of vegetables and only one serve of fruit daily. This could have significant adverse health consequences for women, including an increased risk of obesity [9, 39], cardiovascular diseases and all-cause mortality [40].

Our study found enduring maternal factors associated with following the low versus the high DGI-2013 or RDGI trajectories. Women who smoked or who completed high school or less had the strongest associations with following the low DGI-2013 or RDGI trajectories compared with the high diet quality trajectories, followed by women who were not married or unemployed. Our results are consistent with existing longitudinal research that shows that factors such as smoking [11, 13, 41], lower educational attainment [11, 35, 41–43] and being single [13, 43, 49] are associated with a poorer diet quality in pregnancy and postpartum. Given the consistency in findings regarding lifestyle factors, this indicates that dietary interventions in pregnancy could be complemented by interventions targeting lifestyle, which have shown to be successful in implementing behavioural change [44]. The association between unemployment status and higher odds of following a low versus a high RDGI diet quality trajectory in the current study conflicts with previous longitudinal research, which found no association between employment status and diet quality [13, 41, 43]. The discrepant findings could be due to study design, variability in the timing of employment status assessment (e.g., <18 weeks gestation [13], childbirth [41]) or variability in how participants interpreted employment questions [41].

Maternal age, country of birth and household composition were not associated with the low RDGI and DGI-2013 trajectories. While the null finding for household composition aligns with a prior study of 196 pregnant women from New Zealand [45], the findings for maternal age and country of birth are inconsistent with the literature [32, 43, 46, 47]. The conflicting findings could be ascribed to differences in study design or sample characteristics such as acculturation [48]. Alternatively, the present study used data from an intervention study with less variation in maternal age, country of birth and household composition, which may lead to null findings. No associations were found between pre-pregnancy BMI and diet quality trajectories, which contrasts with recent longitudinal research that demonstrated women with a higher pre-pregnancy BMI were more likely to have a poor diet quality trajectory from pre-pregnancy to 8–9 years postpartum [11]. The null findings in our study could be due to misreporting, which has been found to be common among pregnant women, particularly women with overweight or obesity [49].

Our study found similar effect sizes for the DGI-2013 and RDGI indices despite differences in the index components and the modifications made. This finding suggests that diet quality trajectories were comparable regardless of the diet quality index (with varying number of components) used. The DGI-2013 was chosen for use in this study, as it has been used extensively in the Australian population [8, 24, 50, 51], and the RDGI because of alignment between this study’s dietary assessment tool and the RDGI’s components. Other diet quality indices were considered applicable to this study’s sample of Australian women, such as the Australian Recommended Food Score [52], but could not be applied to the dietary assessment tool used in this study. Thus, diet quality indices that can calculate diet quality scores from brief and long dietary assessment tools are needed. Moreover, no diet quality indices were found for use in pregnant and lactating Australian women. Diet quality indices, such as the Diet Quality Index for Pregnancy for use in pregnant and lactating women from the United States [35], are needed to address this gap.

When participants with missing data were included in the derivation of the diet quality trajectories and the analysis, the results showed consistent but weaker associations. This could be because of differences in maternal background characteristics or diet quality scores. Women with missing data for the maternal factors were more likely to have lower RDGI scores at year 2 compared with women include in the primary analysis. These lower scores could have contributed to the weaker associations observed.

Meeting dietary recommendations in pregnancy and postpartum is vital to avoid complications in pregnancy and postpartum and to ensure good maternal health outcomes [53]. Further, there is a growing body of evidence linking poor maternal nutrition with severe adverse health outcomes in offspring that begin in utero and extend well into adulthood [54]. The findings from our study have the potential to inform dietary intervention strategies by identifying at-risk women who require nutrition intervention. Our study’s results suggest that interventions should begin in pregnancy to have the most benefit. In addition, these interventions should seek to target women identified as at high risk of a poor diet quality, including those who smoke and completed high school or less. Prior research has shown that dietary interventions in pregnancy can trigger healthy eating behaviours in women of diverse socioeconomic backgrounds [55, 56].

The present study has several strengths and limitations. The longitudinal design with four repeated measurements of maternal diet quality provided insight into how maternal diet quality changes over time. In addition, it allowed for an investigation into associations between maternal diet quality trajectories and associated factors and inference of the temporal order of those associations. In addition, using a GBTM approach enabled the inclusion of all available data of participants with two time points, reducing selection and attrition bias [30]. Multiple imputation was conducted to test for potential bias due to missing data, with the results showing consistent but slightly weaker associations. Despite the excluded sample comprising a higher proportion of women with lower educational attainment, which would likely have strengthened the observed associations, our study used data from the Healthy Beginnings Trial, which includes an evenly distributed proportion of women from different educational backgrounds, allowing for the examination of associations between varying levels of educational attainment and diet quality trajectories.

Because of its observational design, our study cannot infer causal relationships. In addition, we used data from a randomised controlled trial; thus, the findings from our study cannot be generalised to the broader population. The dietary assessment tool used in the Healthy Beginnings Trial was relatively brief, and several components and subcomponents from the DGI-2013 and RDGI indices were omitted to calculate the diet quality scores. Because of the omissions made to the two indices used in our analysis, direct comparison of our results with other research using similar diet quality scores might be challenging [57]. For the dairy and alternatives component of the DGI-2013, the FFQ included questions on milk intake only. Therefore, the score for this component might not accurately reflect the participants’ dairy consumption. Data were not collected on the women’s particular week of gestation; thus, it was not possible to include women’s trimester of pregnancy as a covariate.

## Conclusions

This longitudinal analysis provides evidence that diet quality was relatively stable from pregnancy to 3.5 years postpartum, suggesting that dietary behaviours are already established when women are pregnant. Women who smoked or completed high school or less were more likely to follow a low diet quality trajectory. In addition, women who were unemployed or not married were also more likely to have enduring poor diet quality during this period. Together, these findings suggest that efforts to improve diet quality in women should begin in pregnancy, or preferably before pregnancy [11], and it is recommended that public health strategies promoting maternal and child nutrition should be tailored to families most at risk of having a poor diet quality. These strategies have the potential to improve maternal dietary behaviours, leading to positive health outcomes for both mothers and their children. Future research should explore maternal diet quality trajectories from the pre-pregnancy period to determine when dietary interventions should start and understand which maternal factors continue to be associated with poor diet quality over time.

### Electronic supplementary material

Below is the link to the electronic supplementary material.

Supplementary Material 1

## Data Availability

The data supporting this study’s findings are available on request from the corresponding author (MS-D). The data are not publicly available, as they contain information that could compromise research participant privacy.
