# Supplementary material for: Maternal diet quality trajectories from pregnancy to 3.5 years postpartum and associated maternal factors
Source: Eur J Nutr. 2024 May 28;63(5):1961–72. doi: 10.1007/s00394-024-03402-1 (PMC11329599; doi:10.1007/s00394-024-03402-1)
Supplement: Supplementary file 1 — Supplementary Material 1 [file 394_2024_3402_MOESM1_ESM.docx]

# Additional File 1

Supplementary Table 1. STROBE-nut: An extension of the STROBE statement for nutritional epidemiology

Lachat C et al. (2016) STrengthening the Reporting of OBservational studies in Epidemiology – Nutritional Epidemiology (STROBE-nut): an extension of the STROBE statement. Plos Medicine 13(6) <http://dx.doi.org/10.1371/journal.pmed.1002036> [pdf](http://journals.plos.org/plosmedicine/article/asset?id=10.1371%2Fjournal.pmed.1002036.PDF) or [online](http://journals.plos.org/plosmedicine/article?id=10.1371/journal.pmed.1002036) version.

| **Item** | **Item nr** | **STROBE recommendations** | **Extension for Nutritional Epidemiology studies (STROBE-nut)** | **Reported on page #** |
| --- | --- | --- | --- | --- |
| **Title and**  **abstract** | 1 | (a) Indicate the study’s design with a commonly used term in the title or the abstract.  (b) Provide in the abstract an informative and balanced summary of what was done and what was found. | **nut-1** State the dietary/nutritional assessment method(s) used in the title, abstract, or keywords. | **1, 2** |
| **Introduction** |  |  |  |  |
| Background rationale | 2 | Explain the scientific background and rationale for the investigation being reported. |  | **3–4** |
| Objectives | 3 | State specific objectives, including any pre-specified hypotheses. |  | **4** |
| **Methods** |  |  |  |  |
| Study design | 4 | Present key elements of study design early in the paper. |  | **4** |
| Settings | 5 | Describe the setting, locations, and relevant dates, including periods of recruitment, exposure, follow-up, and data collection. | **nut-5** Describe any characteristics of the study settings that might affect the dietary intake or nutritional status of the participants, if applicable. | **4–5** |
| Participants | 6 | a) Cohort study—Give the eligibility criteria, and the sources and methods of selection of participants. Describe methods of follow-up.  Case-control study—Give the eligibility criteria, and the sources and methods of case ascertainment and control selection. Give the rationale for the choice of cases and controls.  Cross-sectional study—Give the eligibility criteria, and the sources and methods of selection of participants.  (b) Cohort study—For matched studies, give matching criteria and number of exposed and unexposed.  Case-control study—For matched studies, give matching criteria and the number of controls per case. | **nut-6** Report particular dietary, physiological or nutritional characteristics that were considered when selecting the target population. | **4** |
| Variables | 7 | Clearly define all outcomes, exposures, predictors, potential confounders, and effect modifiers. Give diagnostic criteria, if applicable. | **nut-7.1** Clearly define foods, food groups, nutrients, or other food components.  **nut-7.2** When using dietary patterns or indices, describe the methods to obtain them and their nutritional properties. | **5–8** |
| Data sources - measurements | 8 | For each variable of interest, give sources of data and details of methods of assessment (measurement).Describe comparability of assessment methods if there is more than one group. | **nut-8.1** Describe the dietary assessment method(s), e.g., portion size estimation, number of days and items recorded, how it was developed and administered, and how quality was assured. Report if and how supplement intake was assessed.  **nut-8.2** Describe and justify food composition data used. Explain the procedure to match food composition with consumption data. Describe the use of conversion factors, if applicable.  **nut-8.3** Describe the nutrient requirements, recommendations, or dietary guidelines and the evaluation approach used to compare intake with the dietary reference values, if applicable.  **nut-8.4** When using nutritional biomarkers, additionally use the STROBE Extension for Molecular Epidemiology (STROBE-ME). Report the type of biomarkers used and their usefulness as dietary exposure markers.  **nut-8.5** Describe the assessment of nondietary data (e.g., nutritional status and influencing factors) and timing of the assessment of these variables in relation to dietary assessment.  **nut-8.6** Report on the validity of the dietary or nutritional assessment methods and any internal or external validation used in the study, if applicable. | **5–6** |
| Bias | 9 | Describe any efforts to address potential sources of bias. | **nut-9** Report how bias in dietary or nutritional assessment was addressed, e.g., misreporting, changes in habits as a result of being measured, or data imputation from other sources | **8, 13–14** |
| Study Size | 10 | Explain how the study size was arrived at. |  | **8–9** |
| Quantitative variables | 11 | Explain how quantitative variables were handled in the analyses. If applicable, describe which groupings were chosen and why. | **nut-11** Explain categorization of dietary/nutritional data (e.g., use of N-tiles and handling of nonconsumers) and the choice of reference category, if applicable. | **7** |
| Statistical  Methods | 12 | (a) Describe all statistical methods, including those used to control for confounding  (b) Describe any methods used to examine subgroups and interactions.  (c) Explain how missing data were addressed.  (d) Cohort study—If applicable, explain how loss to follow-up was addressed.  Case-control study—If applicable, explain how matching of cases and controls was addressed.  Cross-sectional study—If applicable, describe analytical methods taking account of sampling strategy.  (e) Describe any sensitivity analyses. | **nut-12.1** Describe any statistical method used to combine dietary or nutritional data, if applicable.  **nut-12.2** Describe and justify the method for energy adjustments, intake modeling, and use of weighting factors, if applicable.  **nut-12.3** Report any adjustments for measurement error, i.e,. from a validity or calibration study. | **7–8** |
| **Results** |  |  |  |  |
| Participants | 13 | (a) Report the numbers of individuals at each stage of the study—e.g., numbers potentially eligible, examined for eligibility, confirmed eligible, included in the study, completing follow-up, and analyzed.  (b) Give reasons for non-participation at each stage.  (c) Consider use of a flow diagram. | **nut-13** Report the number of individuals excluded based on missing, incomplete or implausible dietary/nutritional data. | **8–9** |
| Descriptive data | 14 | (a) Give characteristics of study participants (e.g., demographic, clinical, social) and information on exposures and potential confounders  (b) Indicate the number of participants with missing data for each variable of interest  (c) Cohort study—Summarize follow-up time (e.g., average and total amount) | **nut-14** Give the distribution of participant characteristics across the exposure variables if applicable. Specify if food consumption of total population or consumers only were used to obtain results. | **9, 20–21** |
| Outcome data | 15 | Cohort study—Report numbers of outcome events or summary measures over time.  Case-control study—Report numbers in each exposure category, or summary measures of exposure.  Cross-sectional study—Report numbers of outcome events or summary measures. |  | **9** |
| Main results | 16 | (a) Give unadjusted estimates and, if applicable, confounder-adjusted estimates and their precision (e.g., 95% confidence interval).  Make clear which confounders were adjusted for and why they were included.  (b) Report category boundaries when continuous variables were categorized.  (c) If relevant, consider translating estimates of relative risk into absolute risk for a meaningful time period. | **nut-16** Specify if nutrient intakes are reported with or without inclusion of dietary supplement intake, if applicable. | **10** |
| Other analyses | 17 | Report other analyses done—e.g., analyses of subgroups and interactions and sensitivity analyses. | **nut-17** Report any sensitivity analysis (e.g., exclusion of misreporters or outliers) and data imputation, if applicable. | **10–11** |
| **Discussion** |  |  |  |  |
| Key results | 18 | Summarize key results with reference to study objectives. |  | **11** |
| Limitation | 19 | Discuss limitations of the study, taking into account sources of potential bias or imprecision. Discuss both direction and magnitude of any potential bias. | **nut-19** Describe the main limitations of the data sources and assessment methods used and implications for the interpretation of the findings. | **13–14** |
| Interpretation | 20 | Give a cautious overall interpretation of results considering objectives, limitations, multiplicity of analyses, results from similar studies, and other relevant evidence. | **nut-20** Report the nutritional relevance of the findings, given the complexity of diet or nutrition as an exposure. | **11–13** |
| Generalizability | 21 | Discuss the generalizability (external validity) of the study results. |  | **13** |
| **Other information** |  |  |  |  |
| Funding | 22 | Give the source of funding and the role of the funders for the present study and, if applicable, for the original study on which the present article is based. |  | **14** |
| *Ethics* |  |  | **nut-22.1** Describe the procedure for consent and study approval from ethics committee(s). | **4, 14–15** |
| *Supplementary material* |  |  | **nut-22.2** Provide data collection tools and data as online material or explain how they can be accessed. | **Additional file 1** |

Supplementary Table 2. Healthy Beginnings Trial survey questions and response options

| **Survey question** | **Response options** |
| --- | --- |
| How many serves of vegetables do you usually eat each day? | Serves per day/per week/don’t eat/don’t know/refused |
| How many serves of fruit do you usually eat each day? | Serves per day/per week/don’t eat/don’t know/refused |
| How often do you usually eat bread? | Times per day/per week/per month/rarely or never/don’t know/refused |
| How many slices of bread do you usually eat each day? | Slices per day/per week/don’t eat/don’t know/refused |
| How often do you eat breakfast cereal? | Times per day/per week/per month/rarely or never/don’t know/refused |
| How often do you eat pasta, rice, noodles or other cooked cereals? | Times per day/per week/per month/rarely or never/don’t know/refused |
| How many cups of milk do you usually drink in a day? | Cups per day/per week/per month/don’t drink/don’t know/refused |
| What type of milk do you usually have? | Regular/low or reduced fat/skim/evaporated or sweetened/reduced fat soy/other/don’t drink/don’t know/refused |
| How often do you eat processed meat products such as sausages, frankfurts, devon, salami, meat pies, bacon or ham? | Times per day/per week/per month/rarely or never/don’t know/refused |
| How often do you have meals or snacks such as burgers, pizza, chicken, or chips from places like McDonalds, Hungry Jacks, Pizza Hut, KFC, Red Rooster or local takeaway food places? | Times per week/per month/rarely or never/don’t know/refused |
| How often do you eat chips, French fries, wedges, fried potatoes or crisps? | Times per day/per week/per month/rarely or never/don’t know/refused |
| How many cups of soft drink, cordial, or sports drink, such as lemonade or Gatorade, do you usually drink in a day? | Cups per day/per week/per month/don’t drink/don’t know/refused |
| How many cups of diet soft drink or diet cordial, such as Diet Coke or Zero Sprite do you usually drink in a day? | Cups per day/per week/per month/don’t drink/don’t know/refused |
| How many cups of fruit juice do you usually drink in a day? | Cups per day/per week/per month/don’t drink/don’t know/refused |
| How many cups of water do you usually drink in a day? | Cups per day/per week/per month/don’t drink/don’t know/refused |

Supplementary Table 3. Modified 2013 Dietary Guideline Index (DGI-2013) [1](1) components, scoring criteria and mapped dietary questions from the Healthy Beginnings Trial

| **Dietary guideline** | **Indicator** | **Max. score criteria ^a^** | **Min. score criteria ^a^** | **Max. possible score ^a^** | **Mapped Healthy Beginnings Trial survey question** |
| --- | --- | --- | --- | --- | --- |
| **Guidelines for adequate intake** | | | | |  |
| 1. Plenty of vegetables | Total serves of vegetables per day | ≥5 serves/day | 0 | 10 | How many serves of vegetables do you usually eat each day? |
| 2. Fruit | Total serves of fruit per day | ≥2 serves/day | 0 | 10 | How many serves of fruit do you usually eat each day? |
| 3. Grain/cereal foods | Total serves of grains per day | ≥6 serves/day | 0 | 5 | How often do you usually eat bread?  How many slices of bread do you usually eat each day?  How often do you eat breakfast cereal?  How often do you eat pasta, rice, noodles or other cooked cereals? |
| 4. Dairy and alternatives ^b^ | Total dairy and alternative serves per day | ≥2.5 serves/day | 0 | 10 | How many cups of milk do you usually drink in a day? |
| 5. Drink plenty of water ^c^ | Total beverage serves per day | ≥8 serves/day | 0 | 5 | How many cups of diet soft drink or diet cordial, such as Diet Coke or Zero Sprite do you usually drink in a day?  How many cups of fruit juice do you usually drink in a day?  How many cups of water do you usually drink in a day? |
|  | Water (proportion of water to total beverage intake per day) | ≥50% | 0% | 5 | How many cups of water do you usually drink in a day? |
| **Guidelines to limit or moderate intake** | | | | |  |
| 6. Limit foods containing saturated fat, added salt, added sugars and alcohol ^d^ | Limit discretionary foods | ≤2.5 serves/day | >2.5 | 10 | How often do you eat processed meat products such as sausages, frankfurts, devon, salami, meat pies, bacon or ham?  How often do you have meals or snacks such as burgers, pizza, chicken, or chips from places like McDonalds, Hungry Jacks, Pizza Hut, KFC, Red Rooster or local takeaway food places?  How often do you eat chips, French fries, wedges, fried potatoes or crisps? |
| 7. Limit foods high in saturated fat ^e^ | Choose reduced-fat milk (type of milk consumed) | Skim, low or reduced-fat milk | Whole milk (0) | Low fat/reduced fat (2.5)  Skim milk (5) | What type of milk do you usually have? |
| 8. Limit foods and drinks containing added sugars ^d^ | Limit extra sugar: serves per day | ≤1.25 serves/day | >1.25 | 10 | How many cups of soft drink, cordial, or sports drink, such as lemonade or Gatorade, do you usually drink in a day? |
| **Total possible score** | | | | **70** |  |

Notes. ^a^ Scoring is based on the national recommendations from the Australian Guide to Healthy Eating for women aged 19–50 years [2]. Scoring was proportional; women whose intakes fell between the minimum and maximum criteria were assigned a proportionate score, and women who met the national recommendations were assigned a maximum score. ^b^ The FFQ contained one question on diary intake related to milk intake that was used to calculate the dairy and alternatives component. ^c^ Water, fruit juice and diet soft drink were included in the calculation of total beverage intake and the proportion of water to total beverages [1,3]. ^d^ The criteria for discretionary foods and added sugars are given as an upper limit. The upper limit was determined as half of the extras food guideline (serves) given that there is no quantitative guideline for added sugars; this is consistent with existing dietary indices [3]. ^e^ The scoring of the saturated fat component was categorical, where a score of 0 was assigned to women who reported consuming whole milk, a score of 2.5 was assigned to women who reported consuming low-fat or reduced-fat milk and a score of 5 was assigned to women who reported drinking skim milk.

Supplementary Table 4. Modified RESIDential Environments Dietary Guideline Index (RDGI) [4](4) components and scoring criteria

| **Component** | **RESIDE indicator** | **Criterion for min. score ^a^** | **Criterion for intermediate score ^a^** | **Criterion for max. score ^a^** |
| --- | --- | --- | --- | --- |
| Vegetables | How many serves of vegetables do you usually eat each day (including fresh, frozen and tinned)? | Do not eat = 0 | >0 and ≤1 serve = 2.5  2 serves = 5  3-4 serves = 7.5 | ≥5 serves = 10 |
| Fruit | How many serves of fruit do you usually eat each day (including fresh, dried, frozen and tinned fruit)? | Do not eat = 0 | >0 and ≤1 serve = 5 | ≥2 serves = 10 |
| Grains/cereals: mostly wholegrain/high fibre | How often do you eat bread (including bread rolls, flat breads, crumpets, bagels, English or bread type muffins)? | < Once per month = 0 | Once per month = 0.5  2–3 times per month = 1  1–2 times per week = 1.5  3–5 times per week = 2 | 6–7 times per week = 2.5 |
|  | How often do you eat pasta, rice, noodles or other cooked cereals? | < Once per month = 0 | Once per month = 1  2–3 times per month = 2  1–2 times per week = 3  3–5 times per week = 4 | 6–7 times per week = 5 |
| Dairy or alternatives: mostly reduced fat | About how much milk (in total) do you usually have in a day? ^b^ | <150 mL = 0 | 150–300 mL = 1.25 | ≥301 mL = 2.5 |
|  | What type of milk do you usually consume? | If whole (full cream) = 0 | If low or reduced fat/other = 1.25 | If skim = 2.5 |
| Drink plenty of water ^c^ | How many cups of water, including sparkling water, do you drink in a day? | Total beverage intake zero cups = 0 | Total beverage intake: 1–7 cups = 2.5 | Total beverage intake: ≥8 cups = 5 |
|  | How many cups of diet or sugar-free soft drinks, cordial or sports drinks do you drink in a day? (such as coke zero or sugar free Gatorade) |  |  |  |
|  | Proportion of water to total beverage intake | 0% = 0 | >0% <50% = 2.5 | ≥50% = 5 |
| Limit intake of foods high in saturated fat | How often do you eat chips, French fries, wedges, fried potatoes or crisps? | 6–7 times per week = 0 | 3–5 times per week = 0.5 1–2 times per week = 1 2–3 times per month = 1.5 | ≤ Once per month = 2 |
|  | How often do you eat meat products such as sausages, frankfurters, polony, meat pies, bacon or ham? | 6–7 times per week = 0 | 3–5 times per week = 0.5 1–2 times per week = 1 2–3 times per month = 1.5 | ≤ Once per month = 2 |
|  | How often do you eat fried, roast or BBQ chicken, pizza, burgers or fish and chips? | 6–7 times per week = 0 | 3–5 times per week = 0.5 1–2 times per week = 1 2–3 times per month = 1.5 | ≤ Once per month = 2 |
| Limit intake of foods and drinks containing added sugars ^d^ | How many cups of regular or sugar sweetened soft drinks, cordial, fruit juice or sports drinks do you drink in a day? | >2 cups = 0 | 1.5–2 cups = 2.5 | ≤1 cup = 5  Don’t drink = 5 |
| **Total possible score** | | | | **53.5** |

Notes. ^a^ Scoring is based on the national recommendations from the Australian Guide to Healthy Eating for women aged 19–50 years [2]. Scoring was proportional and explicitly defined in the dietary index; women were assigned a score if their intake fell within the ranges defined in the index. A maximum score was given to women who were considered to have met the national recommendations. ^b^ Women’s responses for milk intake were given in cups per day, which was considered 250 mL. ^c^ Water and diet soft drink were included in the calculation of total beverage intake and the proportion of water to total beverage intake [4]. ^d^ The criteria for discretionary foods and added sugars are given as an upper limit. The upper limit was determined as half of the extras food guideline (serves) given that there is no quantitative guideline for added sugars; this is consistent with existing dietary indices [3]. Fruit juice and regular soft drink were used to calculate the added sugar component [4].

Supplementary Table 5. Differences in modified 2013 Dietary Guideline Index (DGI-2013) [1] score and modified RESIDential Environments Dietary Guideline Index (RDGI) [4] score by intervention allocation (*n* = 485)

|  | **Control group Mean (SD)** | **Intervention group Mean (SD)** | **Mean difference (95% CI)** | ***p*** |
| --- | --- | --- | --- | --- |
| RDGI score |  |  |  |  |
| Pregnancy (24–34-weeks gestation) | 36.3 (6.2) | 36.0 (6.0) | 0.35 (–0.75, 1.44) | 0.53 |
| Year 1 | 34.6 (7.0) | 35.2 (6.9) | –0.59 (–1.86, 0.67) | 0.36 |
| Year 2 | 35.4 (6.2) | 36.6 (6.5) | –1.20 (–2.35, –0.06) | 0.04 |
| Year 3.5 | 36.0 (5.7) | 35.5 (5.7) | 0.53 (–0.58, 1.64) | 0.35 |
| DGI-2013 score |  |  |  |  |
| Pregnancy (24–34-weeks gestation) | 48.2 (9.4) | 47.5 (9.1) | 0.74 (–0.91, 2.39) | 0.38 |
| Year 1 | 43.4 (10.0) | 44.3 (9.8) | –0.87 (–2.68, 0.94) | 0.35 |
| Year 2 | 45.1 (8.7) | 46.1 (8.8) | –0.97 (–2.55, 0.60) | 0.23 |
| Year 3.5 | 46.2 (7.2) | 46.0 (7.2) | 0.18 (–1.22, 1.57) | 0.80 |

Notes. Differences were assessed using the *t*-test. CI, confidence interval; DGI-2013, 2013 Dietary Guideline Index; IQR, interquartile range; RDGI, RESIDential Environments Dietary Guideline Index.

Supplementary Table 6. Pearson correlation matrix of correlations between maternal factors (n = 485)

|  | **Age** | **Country of birth** | **Educational attainment** | **Employment status** | **Household income** | **Marital status** | **Household composition** | **Smoking status** | **Pre-pregnancy BMI** |
| --- | --- | --- | --- | --- | --- | --- | --- | --- | --- |
| **Age** | 1.0000 |  |  |  |  |  |  |  |  |
| **Country of birth** | –0.1783 | 1.0000 |  |  |  |  |  |  |  |
| **Educational attainment** | 0.3740 | –0.2140 | 1.0000 |  |  |  |  |  |  |
| **Employment status** | –0.2964 | –0.1708 | –0.2237 | 1.0000 |  |  |  |  |  |
| **Household income** | 0.3789 | 0.0604 | 0.3704 | –0.5090 | 1.0000 |  |  |  |  |
| **Marital status** | 0.2772 | –0.1553 | 0.2331 | –0.2381 | 0.3832 | 1.0000 |  |  |  |
| **Household composition** | –0.2630 | –0.1048 | –0.1512 | 0.2135 | –0.3169 | –0.4272 | 1.0000 |  |  |
| **Smoking status** | –0.0463 | 0.1904 | –0.2547 | 0.0355 | –0.1198 | –0.1342 | 0.1129 | 1.0000 |  |
| **Pre-pregnancy BMI** | 0.0925 | 0.2722 | –0.0808 | –0.1267 | 0.0597 | 0.0738 | –0.1060 | 0.1041 | 1.0000 |

Notes. Age and pre-pregnancy BMI are continuous variables; all other variables are categorical. BMI, body mass index.

Supplementary Table 7. Characteristics, modified 2013 Dietary Guideline Index (DGI-2013) [1] z-score and modified RESIDential Environments Dietary Guideline Index (RDGI) [4] z-score of complete case analysis sample and excluded sample

| **Characteristic** | **Complete case analysis sample (n = 473)** | | **Excluded sample (n = 194)** | | ***p*** |
| --- | --- | --- | --- | --- | --- |
|  | ***n* (%)** | **Mean (SD)/median (IQR)** | ***n* (%)** | **Mean (SD)/median (IQR)** |  |
| Age, y | 473 (100) | 27.2 (5.2) | 194 (100) | 24.4 (5.7) | <0.0001 |
| Country of birth |  |  |  |  | 0.46 |
| Australia | 301 (63.6) |  | 128 (66.6) |  |  |
| Other | 172 (36.4) |  | 64 (33.3) |  |  |
| Educational attainment |  |  |  |  | <0.0001 |
| High school or less | 159 (33.6) |  | 102 (52.5) |  |  |
| TAFE certificate/diploma | 181 (38.3) |  | 62 (32.0) |  |  |
| University | 133 (28.1) |  | 30 (15.5) |  |  |
| Employment status |  |  |  |  | <0.0001 |
| Employed/maternity leave | 284 (60.0) |  | 79 (40.9) |  |  |
| Unemployed | 189 (40.0) |  | 114 (59.1) |  |  |
| Marital status |  |  |  |  | <0.0001 |
| Not married | 40 (8.5) |  | 43 (22.4) |  |  |
| Married/de facto | 433 (91.5) |  | 149 (77.6) |  |  |
| Household composition |  |  |  |  | 0.001 |
| Two parent | 352 (74.4) |  | 112 (61.2) |  |  |
| Other | 121 (25.6) |  | 71 (38.8) |  |  |
| Smoking status |  |  |  |  | 0.007 |
| Non-smoker | 318 (67.2) |  | 106 (56.1) |  |  |
| Current/past smoker | 155 (32.8) |  | 83 (43.9) |  |  |
| Pre-pregnancy BMI, kg/m^2^ | 448 (94.7) | 23.9 (21.1, 27.6) | 178 (91.8) | 23.1 (20.3, 28.0) | 0.36 |
| DGI-2013 score |  |  |  |  |  |
| Pregnancy (24–34-weeks gestation) | 473 (100) | 48.0 (9.1) | 193 (99.5) | 43.8 (10.7) | <0.0001 |
| 1 year postpartum | 454 (96.0) | 44.0 (9.8) | 73 (37.6) | 40.5 (10.6) | 0.0002 |
| 2 years postpartum | 464 (98.1) | 45.8 (8.6) | 33 (17.0) | 39.8 (10.6) | <0.0001 |
| 3.5 years postpartum | 399 (84.4) | 46.1 (7.1) | 16 (8.2) | 40.5 (10.2) | <0.0001 |
| RDGI score |  |  |  |  |  |
| Pregnancy (24–34-weeks gestation) | 473 (100) | 36.3 (6.1) | 193 (99.5) | 33.3 (6.6) | <0.0001 |
| 1 year postpartum | 454 (96.0) | 35.0 (6.9) | 73 (37.6) | 31.7 (7.6) | 0.0056 |
| 2 years postpartum | 464 (98.1) | 36.2 (6.3) | 33 (17.0) | 31.4 (7.0) | 0.0002 |
| 3.5 years postpartum | 399 (84.4) | 35.8 (5.7) | 16 (8.2) | 29.4 (8.5) | 0.0028 |

Notes. IQR, interquartile rage; SD, standard deviation

Supplementary Table 8. Bayesian information criterion, entropy and proportion of the smallest group of the group-based trajectory modelling (2–5 groups) for the modified 2013 Dietary Guideline Index (DGI-2013) [1] z-score and the modified RESIDential Environments Dietary Guideline Index (RDGI) [4] z-score (n = 485)

| **DGI-2013 z-score** | | | | **RDGI z-score** | | | |
| --- | --- | --- | --- | --- | --- | --- | --- |
| **Number of groups** | **BIC** | **Entropy** | **Smallest group (%)** | **Number of groups** | **BIC** | **Entropy** | **Smallest group (%)** |
| 2 (3,3) | –2452.09 | 0.76 | 33.8 | 2 (3,3) | –2418.64 | 0.76 | 37.9 |
| 3 (3,3,3) | –2440.87 | 0.75 | 5.15 | 3 (3,3,3) | –2402.58 | 0.72 | 9.5 |
| 4 (3,3,3,3) | –2439.31 | 0.64 | 6.6 | 4 (3,3,3,3) | –2403.2 | 0.68 | 8.45 |
| 5 (3,3,3,3,3) | –2442.15 | 0.67 | 4.7 | 5 (3,3,3,3,3) | –2414.58 | 0.67 | 8.66 |

Supplementary Table 9. Univariable and multivariable logistic regression analyses estimating associations between maternal factors and following the low versus the high 2013 Dietary Guideline Index (DGI-2013) [1] or RESIDential Environments Dietary Guideline Index (RDGI) [4] raw score trajectory among pregnant women in the Healthy Beginnings Trial

| Characteristic | Univariable model | | | Multivariable model | | | Univariable model | | | Multivariable model | | |
| --- | --- | --- | --- | --- | --- | --- | --- | --- | --- | --- | --- | --- |
|  | High DGI-2013 trajectory (raw score) | Low DGI-2013 trajectory (raw score) | | High DGI-2013 trajectory (raw score) | Low DGI-2013 trajectory (raw score) | | High RDGI trajectory (raw score) | Low RDGI trajectory (raw score) | | High RDGI trajectory (raw score) | Low RDGI trajectory (raw score) | |
|  | Ref. | OR ^a^ (95% CI) | *p* | Ref. | OR ^a^ (95% CI) | *p* | Ref. | OR ^a^ (95% CI) | *p* | Ref. | OR ^a^ (95% CI) | *p* |
| Age, y |  | 0.91 (0.88, 0.95) | <0.0001 |  | 0.96 (0.92, 1.00) | 0.075 |  | 0.93 (0.89, 0.96) | <0.0001 |  | 0.98 (0.94, 1.02) | 0.35 |
| Country of birth, n (%) | 1 |  |  | 1 |  |  | 1 |  |  | 1 |  |  |
| Other (Ref.) |  |  |  |  |  |  |  |  |  |  |  |  |
| Australia |  | 2.00 (1.30, 3.06) | 0.002 |  | 1.37 (0.84, 2.24) | 0.20 |  | 1.89 (1.26, 2.83) | 0.002 |  | 1.48 (0.92, 2.36) | 0.11 |
| Educational attainment, n (%) | 1 |  |  | 1 |  |  | 1 |  |  | 1 |  |  |
| High school or below (Ref) |  |  |  |  |  |  |  |  |  |  |  |  |
| TAFE certificate/diploma |  | 0.71 (0.46, 1.10) | 0.12 |  | 0.93 (0.58, 1.50) | 0.78 |  | 0.66 (0.43, 1.02) | 0.06 |  | 0.84 (0.53, 1.35) | 0.48 |
| University |  | 0.19 (0.10, 0.35) | <0.0001 |  | 0.35 (0.18, 0.68) | 0.002 |  | 0.20 (0.11, 0.34) | <0.0001 |  | 0.34 (0.18, 0.63) | 0.001 |
| Employment status, n (%) | 1 |  |  | 1 |  |  | 1 |  |  | 1 |  |  |
| Employed (Ref.) |  |  |  |  |  |  |  |  |  |  |  |  |
| Unemployed |  | 1.87 (1.26, 2.78) | 0.002 |  | 1.4 (0.88, 2.23) | 0.15 |  | 2.09 (1.43, 3.08) | <0.0001 |  | 1.76 (1.12, 2.75) | 0.014 |
| Marital status | 1 |  |  | 1 |  |  | 1 |  |  | 1 |  |  |
| Not married (Ref.) |  |  |  |  |  |  |  |  |  |  |  |  |
| Married/de facto |  | 0.21 (0.11, 0.43) | <0.0001 |  | 0.36 (0.15, 0.82) | 0.016 |  | 0.29 (0.14, 0.56) | <0.0001 |  | 0.59 (0.26, 1.36) | 0.22 |
| Household composition | 1 |  |  | 1 |  |  | 1 |  |  | 1 |  |  |
| Other (Ref.) |  |  |  |  |  |  |  |  |  |  |  |  |
| Two-parent household |  | 0.70 (0.46, 1.09) | 0.115 |  | 1.36 (0.77, 2.38) | 0.29 |  | 0.67 (0.44, 1.02) | 0.062 |  | 1.07 (0.63, 1.82) | 0.80 |
| Smoking status | 1 |  |  | 1 |  |  | 1 |  |  | 1 |  |  |
| Non-smoker (Ref.) |  |  |  |  |  |  |  |  |  |  |  |  |
| Current/past smoker |  | 2.25 (1.50, 3.37) | <0.0001 |  | 1.64 (1.05, 2.55) | 0.029 |  | 2.4 (1.62, 3.57) | <0.0001 |  | 1.75 (1.14, 2.69) | 0.011 |

Notes. CI, confidence interval; DGI-2013, 2013 Dietary Guideline Index; OR, odds ratio.

Supplementary Table 10. Univariable and multivariable logistic regression analyses with missing data imputation estimating associations between maternal factors and following the low versus the high 2013 Dietary Guideline Index (DGI-2013) [1] or RESIDential Environments Dietary Guideline Index (RDGI) [4] z-score trajectory among pregnant women in the Healthy Beginnings Trial

| Characteristic | Univariable model | | | Multivariable model | | | Univariable model | | | Multivariable model | | |
| --- | --- | --- | --- | --- | --- | --- | --- | --- | --- | --- | --- | --- |
|  | High DGI-2013 trajectory | Low DGI-2013 trajectory | | High DGI-2013 trajectory | Low DGI-2013 trajectory | | High RDGI trajectory | Low RDGI trajectory | | High RDGI trajectory | Low RDGI trajectory | |
|  | Ref. | OR ^a^ (95% CI) | *p* | Ref. | OR ^a^ (95% CI) | *p* | Ref. | OR ^a^ (95% CI) | *p* | Ref. | OR ^a^ (95% CI) | *p* |
| Age, y |  | 0.92 (0.88, 0.96) | <0.0001 |  | 0.96 (0.91, 1.00) | 0.067 |  | 0.91 (0.88, 0.95) | <0.0001 |  | 0.96 (0.92, 1.00) | 0.069 |
| Country of birth, n (%) | 1 |  |  | 1 |  |  | 1 |  |  | 1 |  |  |
| Other (Ref.) |  |  |  |  |  |  |  |  |  |  |  |  |
| Australia |  | 2.04 (1.35, 3.11) | 0.001 |  | 1.33 (0.81, 2.16) | 0.26 |  | 1.79 (1.20, 2.66) | 0.004 |  | 1.31 (0.81, 2.13) | 0.27 |
| Educational attainment, n (%) | 1 |  |  | 1 |  |  | 1 |  |  | 1 |  |  |
| High school or below (Ref) |  |  |  |  |  |  |  |  |  |  |  |  |
| TAFE certificate/diploma |  | 0.64 (0.41, 0.98) | 0.038 |  | 0.81 (0.51, 1.30) | 0.39 |  | 0.62 (0.40, 0.94) | 0.025 |  | 0.83 (0.52, 1.31) | 0.41 |
| University |  | 0.21 (0.12, 0.37) | <0.0001 |  | 0.40 (0.22, 0.75) | 0.004 |  | 0.20 (0.12, 0.35) | <0.0001 |  | 0.39 (0.21, 0.71) | 0.002 |
| Employment status, n (%) | 1 |  |  | 1 |  |  | 1 |  |  | 1 |  |  |
| Employed (Ref.) |  |  |  |  |  |  |  |  |  |  |  |  |
| Unemployed |  | 1.60 (1.09, 2.35) | 0.016 |  | 1.17 (0.74, 1.84) | 0.50 |  | 2.10 (1.44, 3.06) | <0.0001 |  | 1.63 (1.05, 2.54) | 0.029 |
| Marital status | 1 |  |  | 1 |  |  | 1 |  |  | 1 |  |  |
| Not married (Ref.) |  |  |  |  |  |  |  |  |  |  |  |  |
| Married/de facto |  | 0.21 (0.11, 0.40) | <0.0001 |  | 0.32 (0.15, 0.71) | 0.005 |  | 0.26 (0.14, 0.50) | <0.0001 |  | 0.54 (0.24, 1.18) | 0.12 |
| Household composition | 1 |  |  | 1 |  |  | 1 |  |  | 1 |  |  |
| Other (Ref.) |  |  |  |  |  |  |  |  |  |  |  |  |
| Two-parent household |  | 0.68 (0.45, 1.03) | 0.072 |  | 1.29 (0.75, 2.23) | 0.36 |  | 0.61 (0.41, 0.93) | 0.02 |  | 1.05 (0.62, 1.76) | 0.87 |
| Smoking status | 1 |  |  | 1 |  |  | 1 |  |  | 1 |  |  |
| Non-smoker (Ref.) |  |  |  |  |  |  |  |  |  |  |  |  |
| Current/past smoker |  | 2.28 (1.53, 3.39) | <0.0001 |  | 1.70 (1.10, 2.63) | 0.017 |  | 2.31 (1.56, 3.41) | <0.0001 |  | 1.74 (1.14, 2.68) | 0.011 |
| Pre-pregnancy BMI, kg/m^2^ |  | 1.02 (0.98, 1.05) | 0.31 |  | 1.01 (0.97, 1.05) | 0.58 |  | 1.01 (0.98, 1.05) | 0.47 |  | 1.01 (0.97, 1.05) | 0.70 |

Notes. CI, confidence interval; DGI-2013, 2013 Dietary Guideline Index; OR, odds ratio.


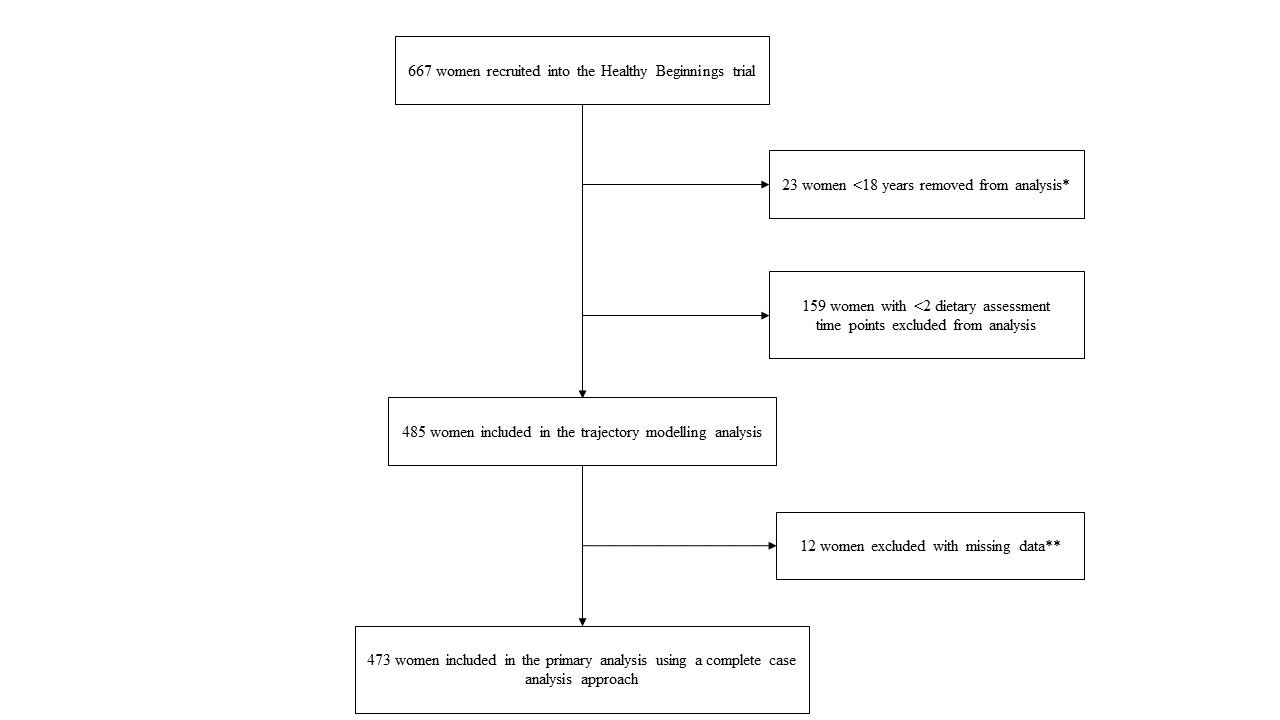


Supplementary Fig. 1 Study flow diagram

Notes. * Women <18 years excluded from the analysis because the RESIDential Environments Dietary Guideline Index (RDGI) was developed for use in adults aged <18 years only [4]. ** Missing data: country of birth, *n* = 2; employment status, *n* = 1; household composition, *n* = 6; smoking status, *n* = 3.


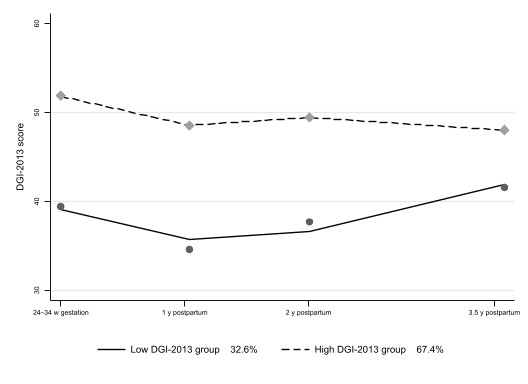


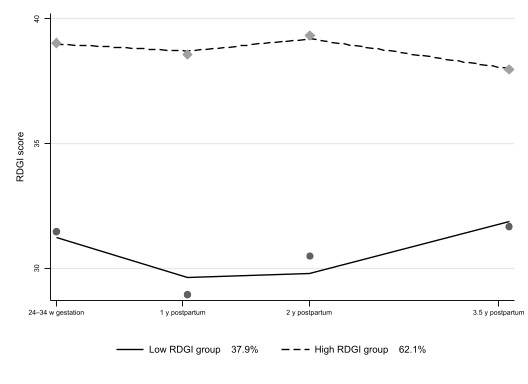


Supplementary Figure 2. 2013 Dietary Guideline Index (DGI-2013) [1] and RESIDential Environments Dietary Guideline Index (RDGI) [4] Raw Score Trajectories from Pregnancy (24–34-Weeks Gestation) to 3.5 Years Postpartum.

Notes. DGI-2013, 2013 Dietary Guideline Index; RDGI, RESIDential Environments Dietary Guideline Index; SD, standard deviation; w, weeks; y, year(s).

## References

1. Thorpe MG, Milte CM, Crawford D et al. (2016) A revised Australian Dietary Guideline Index and its association with key sociodemographic factors, health behaviors and body mass index in peri-retirement aged adults. Nutrients 8(3):160
2. National Health and Medical Research Council. (2013) Eat for health. Australian dietary guidelines summary. National Health and Medical Research Council, Canberra
3. McNaughton SA BK, Crawford D, Mishra GD. (2008) An index of diet and eating patterns is a valid measure of diet quality in an Australian population. J Nutr 138(1):86–93
4. Bivoltsis A, Trapp GSA, Knuiman M et al. (2018) Can a simple dietary index derived from a sub-set of questionnaire items assess diet quality in a sample of Australian adults? Nutrients 10(4):486
